# Integration of adeno-associated virus (AAV) into the genomes of most Thai and Mongolian liver cancer patients does not induce oncogenesis

**DOI:** 10.1186/s12864-021-08098-9

**Published:** 2021-11-11

**Authors:** Alejandro A. Schäffer, Dana A. Dominguez, Lesley M. Chapman, E. Michael Gertz, Anuradha Budhu, Marshonna Forgues, Jittiporn Chaisaingmongkol, Siritida Rabibhadana, Benjarath Pupacdi, Xiaolin Wu, Enkhjargal Bayarsaikhan, Curtis C. Harris, Mathuros Ruchirawat, Eytan Ruppin, Xin Wei Wang

**Affiliations:** 1 Cancer Data Science Laboratory, Center for Cancer Research, National Cancer Institute, National Institutes of Health, Bethesda, MD USA; 2 Laboratory of Human Carcinogenesis, Center for Cancer Research, National Cancer Institute, National Institutes of Health, Bethesda, MD USA; 3 Liver Cancer Program, Center for Cancer Research, National Cancer Institute, National Institutes of Health, Bethesda, MD USA; 4 Laboratory of Chemical Carcinogenesis, Chulabhorn Research Institute, Bangkok, Thailand; 5 Center of Excellence on Environmental Health and Toxicology, Office of Higher Education Commission, Ministry of Education, Bangkok, Thailand; 6 Frederick National Laboratory for Cancer Research, Leidos Biomedical Research Inc, Frederick, MD USA; 7 General Laboratory Department, National Cancer Center, Ulaanbaatar, Mongolia

**Keywords:** Liver cancer, Hepatocellular carcinoma, Intrahepatic cholangiocarcinoma, Adeno-associated virus, Virus integration, Viral oncogenesis, Viral capture sequencing, Gene therapy, Sequence analysis

## Abstract

**Background:**

Engineered versions of adeno-associated virus (AAV) are commonly used in gene therapy but evidence revealing a potential oncogenic role of natural AAV in hepatocellular carcinoma (HCC) has raised concerns. The frequency of potentially oncogenic integrations has been reported in only a few populations. AAV infection and host genome integration in another type of liver cancer, cholangiocarcinoma (CCA), has been studied only in one cohort. All reported oncogenic AAV integrations in HCC come from strains resembling the fully sequenced AAV2 and partly sequenced AAV13. When AAV integration occurs, only a fragment of the AAV genome is detectable in later DNA or RNA sequencing. The integrated fragment is typically from the 3’ end of the AAV genome, and this positional bias has been only partly explained. Three research groups searched for evidence of AAV integration in HCC RNAseq samples in the Cancer Genome Atlas (TCGA) but reported conflicting results.

**Results:**

We collected and analyzed whole transcriptome and viral capture DNA sequencing in paired tumor and non-tumor samples from two liver cancer Asian cohorts from Thailand (*N* = 147, 47 HCC and 100 intrahepatic cholangiocarcinoma (iCCA)) and Mongolia (*N* = 70, all HCC). We found only one HCC patient with a potentially oncogenic integration of AAV, in contrast to higher frequency reported in European patients. There were no oncogenic AAV integrations in iCCA patients. AAV genomic segments are present preferentially in the non-tumor samples of Thai patients.

By analyzing the AAV genome positions of oncogenic and non-oncogenic integrated fragments, we found that almost all the putative oncogenic integrations overlap the X gene, which is present and functional only in the strain AAV2 among all fully sequenced strains. This gene content difference could explain why putative oncogenic integrations from other AAV strains have not been reported.

We resolved the discrepancies in previous analyses of AAV presence in TCGA HCC samples and extended it to CCA. There are 12 TCGA samples with an AAV segment and none are in Asian patients. AAV segments are present in preferentially in TCGA non-tumor samples, like what we observed in the Thai patients.

**Conclusions:**

Our findings suggest a minimal AAV risk of hepatocarcinogenesis in Asian liver cancer patients. The partial genome presence and positional bias of AAV integrations into the human genome has complicated analysis of possible roles of AAV in liver cancer.

**Supplementary Information:**

The online version contains supplementary material available at 10.1186/s12864-021-08098-9.

## Background

Certain viruses harbor the potential to affect human health by altering host immunity and the genome, potentially contributing to chronic disease and cancer. Previously thought to be non-pathogenic in humans, the adeno-associated virus (AAV) is a DNA virus commonly used as a vector in gene therapy [1, 2]. In European and a few Japanese hepatocellular carcinoma (HCC) patients, it has been shown that AAV strains similar to AAV2 or AAV13 have the potential to integrate into genes including *CCNA2* and *CCNE1*, contributing to oncogenesis [3–6]. Here, we use the notation AAV2/13 because the observed AAV sequences in HCC come from strains between the fully-sequenced AAV2 and the partly-sequenced AAV13[3, 5]. The latter study emphasized that some integrated sequences best match AAV2, while other integrated sequences lie between AAV2 and AAV13 in an AAV phylogenetic tree [5]. Additionally, the risk of insertional oncogenesis for liver cancer has been suggested in animal models of AAV2-based gene therapy [7]. In mouse experiments with gene therapy, at least part of the risk appears to be due to the integration of AAV vectors at a particular locus called *Rian*, for which there is no orthologous locus in the human genome [8, 9]. Recent reports on long-term studies of AAV-based therapy for hemophilia A in dogs [10] and hemophilia B in humans [9] found no cases of liver cancer, but the sample sizes are small.

We examine the prevalence of AAV segment presence and integration in two Asian cohorts including both HCC and intrahepatic cholangiocarcinoma (iCCA) samples. One previous study included 46 cholangiocarcinoma cases among 1461 cases, and among evaluable samples, reported presence of AAV in 7/43 non-tumor samples and 2/45 tumor samples with no evidence of oncogenic integrations [5].

### Past analyses of AAV integration in TCGA liver cancer samples

At least three research groups have previously searched the Cancer Genome Atlas (TCGA) for evidence of AAV but reached quite different conclusions [4, 11, 12]; two other groups also searched for viruses in TCGA, but for reasons partly explained in the next paragraph their methods could not have found AAV [13, 14]. The first study reported oncogenic integrations in four HCC (the TCGA code for HCC is LIHC) tumor samples but did not consider non-tumor samples or CCA samples [4]. The second study identified AAV fragments in six TCGA HCC samples but deemed them to be contamination because the locations in the AAV genome are concentrated at the 3’ end [11]. The third study included the larger Pan-Cancer Analysis of Whole Genomes (PCAWG) sample set and reported only one sample with AAV in the non-TCGA subset of PCAWG without commenting on the discrepancies with previous findings [12].

When AAV integrates into the human genome and sequencing is done later, only fragments of the AAV genome are detectable [5, 15]. Complex AAV integration fragments were also found when hemophelic dogs were treated with a recombinant AAV-derived gene therapy vector [10]. This phenomenon is not unique to AAV and has been seen in other cancer-related viruses; this phenomenon is part of a larger set of virus-host phenomena named “hit-and-run” [16]. In the case of AAV, there may be a long latency between the initial integration and the activation of AAV by a helper virus, such as an adenovirus or a herpesvirus [15, 17]. Because of the combined latency for activation of AAV and latency for development of liver cancer, it has been challenging to identify which helper virus may have activated AAV. In the largest-scale study of liver cancer and AAV to date, a candidate helper virus could be found in fewer than 45 % of the cases [5].

### Strains of AAV and their genes

AAV belongs to the family *Parvoviridae* and the genus *Dependoparvorius.* Our sequence analyses used the following complete genomes occurring in nature: AAV1 (NC_002077.1), AAV2 (NC_001401.2), AAV3 (NC_001729.1), AAV4 (NC_001829.1), AAV5 (NC_006152.1), AAV7 (NC_006260.1), AAV8 (NC_006261.1) and the partial genome of AAV13 (EU285562.1); for additional strains, some strain-specific PCR primers have been tested [18]. We did not include laboratory-engineered AAV sequences, such as HV550988.1 and DD233401.1, because these AAV sequences should not appear in a patient infected outside the laboratory or a gene therapy trial.

**Fig. 1:**
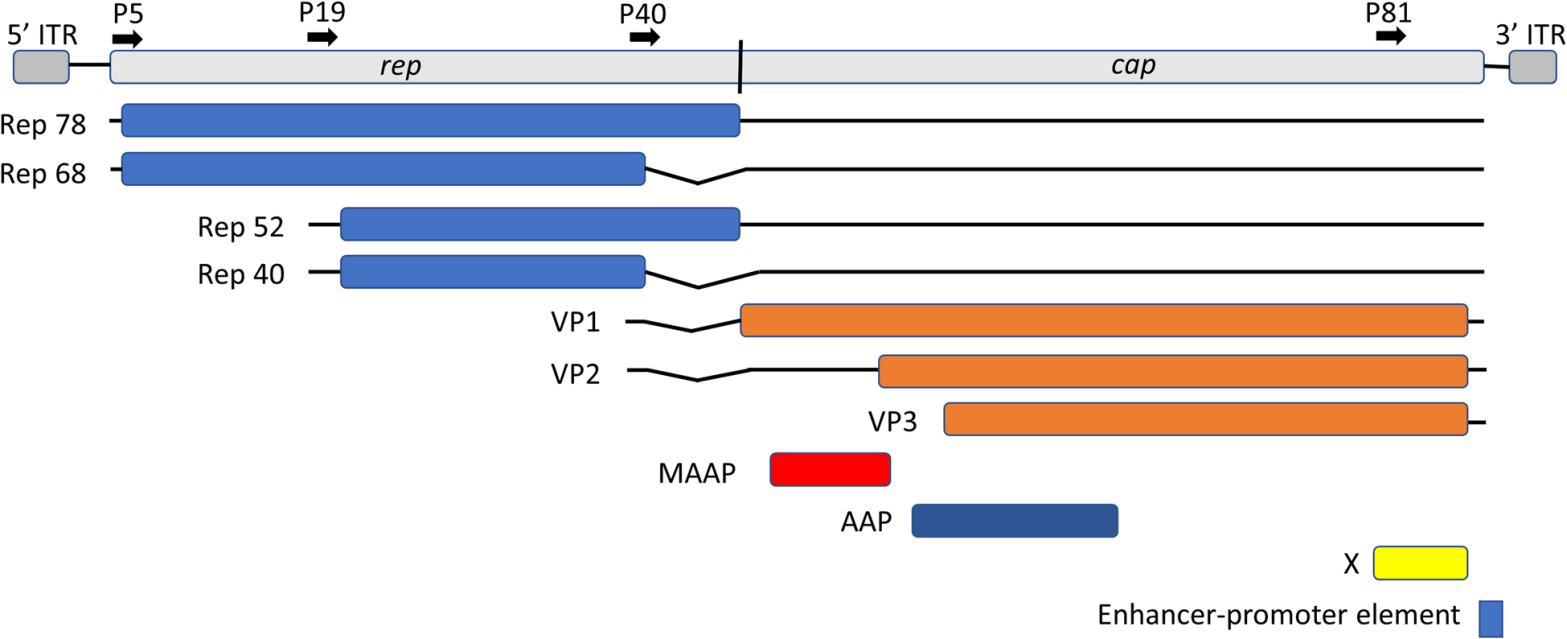
Visualization of the adeno-associated virus strain 2 (AAV2) genome including the X gene [20] and a promoter-enhancer region [36] that may be important to carcinogenesis but are not typically shown in schematic illustrations of AAV2

Typically (e.g., https://viralzone.expasy.org/226?outline=all_by_species), AAV2 is characterized as having seven proteins, comprised of four overlapping non-structural Rep proteins encoded from the N-terminal portion and three overlapping structural proteins VP1, VP2, VP3 encoded from the C-terminal portion. The VP proteins make up the virus capsid and hence are sometimes called “cap” or “capsid” proteins instead of VP. In Fig. 1, we show these open reading frames (ORFs) as well as an additional regulatory region.

At least three other protein coding genes in AAV2 have been reported [19–21]. One of these, called the X gene/protein has sufficient evidence that is explicitly annotated in the GenBank record for NC_001401.2. The X gene spans positions 3,929-4,396 in the reference AAV2 genome. The X gene sequence has been patented based on some evidence for a role in oncogenesis [22, 23]. One of those pieces of evidence is that wild-type AAV2 can replicate autonomously in a keratinocyte tissue culture system [24], but mutants that disrupt X cannot replicate autonomously in that system [22, 23]. This finding shows that although the original name “adeno-associated virus” was meant to imply that coinfection with adenovirus was necessary for replication, coinfection is not necessary in some conditions. In Chinese hamster cells, it was demonstrated in the lab of Harald zur Hausen that autonomous replication of wild type AAV2 is possible [25]. The later tissue culture experiment was in human cells and hence one step closer to the human *in vivo* setting. Another piece of evidence that the X gene is an oncogene is that its presence can lead to transformation of 3T3 cells [23]. The existence of the 155 amino acid X protein (YP_009110690.1) has been validated using a non-targeted set of mRNA and proteomics experiments [26].

All six completely sequenced AAV strains other than AAV2 lack an intact X gene; whether AAV13 contains an intact X gene is unknown. In the first paper about the X gene, Cao and colleagues (see their Table 1) summarized that non-AAV2 strains have sequence segments with weak similarity to the X gene [20], but we checked that none of the alignments are full length. Cao and colleagues went on to show that in the AAV6 strain, there are two separate segments similar to different regions of the AAV2 X gene, and that insertion of the AAV2 X gene into AAV6 significantly improves replication of the modified AAV6 in HEK293 cells compared to wild-type AAV6. From these comparative observations, Cao and colleagues inferred that the X gene is functional and the segments in wild type AAV6 similar to AAV2 are not functional [22].

**Table 1:** Clinical Characteristics of Mongolia and Thailand Cohorts IQR: Interquartile range; HBV: Hepatitis B virus; HCV: Hepatitis C virus; HDV: Hepatitis D virus. AFP: alpha-fetoprotein level. AAV2/13 refers to any sequence that most closely resembles the full AAV2 genome or the partial AAV13 genome but could be phylogenetically in between. Missing data excluded. Entries with a – were not measured

	Mongolia		Thailand (HCC)		Thailand (iCCA)	
	*N or mean*	%	*N or mean*	%	*N or mean*	%
Age (median, IQR)	60	55–66	56.3	51–61	59.5	54–65
BMI (median, IQR)	24.7	21.3–27.9	22.8	20.8–25.3	22.3	19.8–24.4
Smoker	19/62	30.6 %	30/45	66.7 %	55/100	55 %
Drink Alcohol	8/62	12.9 %	37/47	78.7 %	64/100	64 %
HBV (HBsAg)	48/70	68.6 %	22/42	52.4 %	2/84	2.4 %
HCV (HCAb)	40/70	57.1 %	5/40	12.5 %	3/83	3.6 %
HDV (HDAb)	27/70	38.6 %	-	-	-	-
Fam History Liver Cancer	11/61	18.0 %	18/59	30.5 %	41/94	43.6 %
Cirrhosis	27/56	48.2 %	23/36	63.8 %	-	-
AFP (median, IQR)	-	-	38.6	5.8-320.7	-	-
Multinodular	13/61	21.3 %	12/36	33.3 %	3/42	7.1 %
TNM staging						
*Stage I*	1	1.9 %	13	36.1 %	16	40 %
*Stage II*	19	35.8 %	15	41.7 %	5	12.5 %
*Stage III*	31	58.5 %	5	13.9 %	7	17.5 %
*Stage IV*	2	3.8 %	3	8.3 %	12	30 %
AAV2/13 + Tumor RNA-Seq	0	0 %	0	0 %	0	0 %
AAV2/13 + Non-tumor RNA-Seq	2	2.9 %	3	6.4 %	14	14 %
AAV2/13 + Tumor Viral Capture DNA-Seq	0	0 %	1	33.3 %	4	4 %
AAV2/13 + Non-tumor Viral Capture DNA-Seq	0	0 %	3	6.4 %	13	13 %
AAV2/13 + Tumor Genome Insertion	0	0 %	1	2.1 %	2	2 %
AAV2/13 + Non-tumor Genome Insertion	0	0 %	2	4.3 %	9	9 %
AAV2/13 + Shared Genome Insertion	0	0 %	1	2.1 %	0	0 %

### Our contributions

We searched for evidence of AAV in RNASeq and targeted DNASeq samples of two large cohorts of liver cancer patients from Thailand and Mongolia. We did positional analysis of all integrations we found. We also repeated an analysis of AAV presence in TCGA, extending it to CCA (CHOL) and to non-tumor samples, and we resolved the discrepancies mentioned above.

## Results and Discussion

**Fig. 2:**
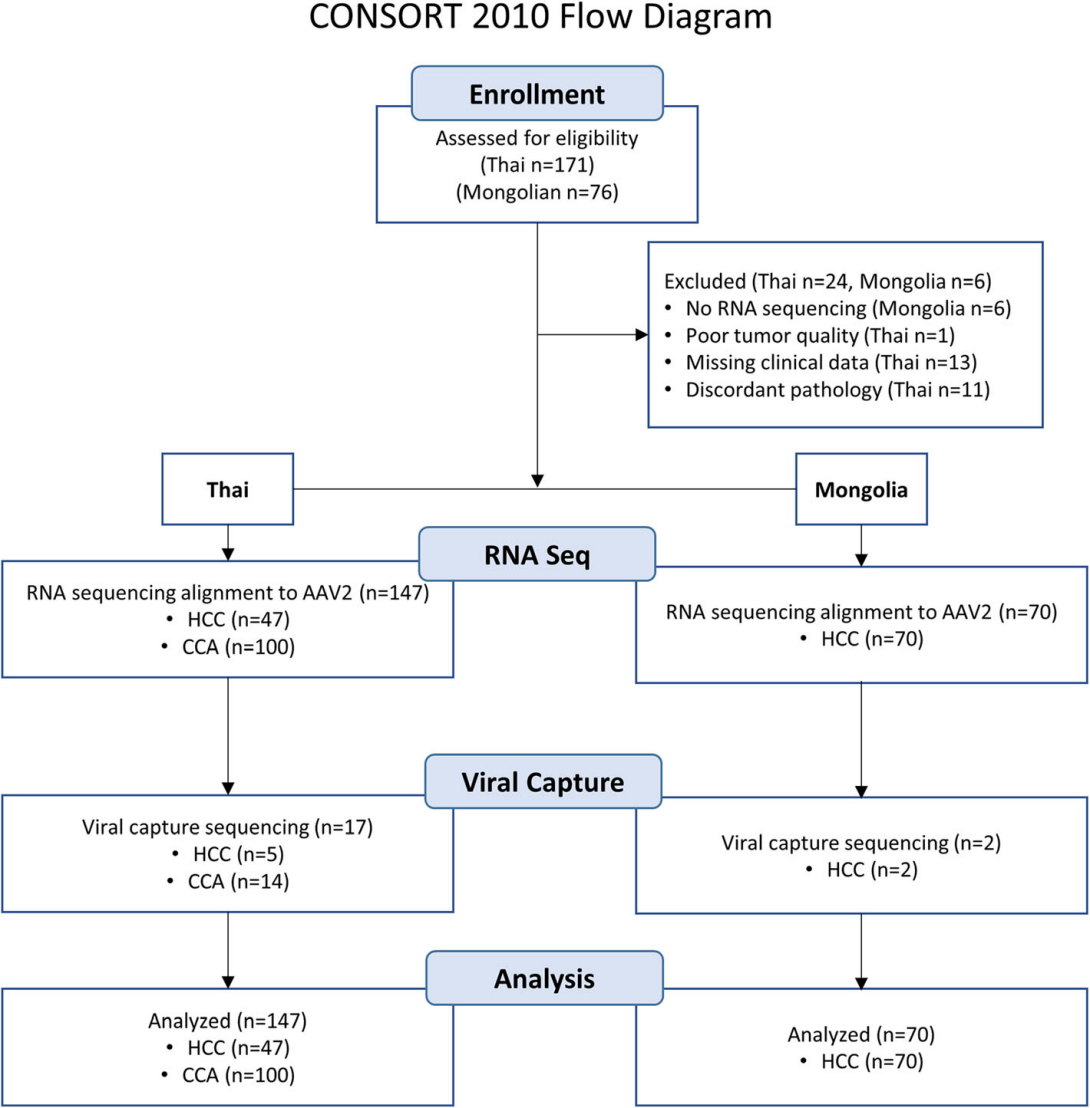
Design of the study and patient selection. The RNA sequencing can detect expression of transcripts containing viral genome segments as explained in the flowchart of Fig. 3 A. The viral capture DNA sequencing was designed to detect AAV sequences that are present and AAV sequences that are integrated into the host genome, whether the sequences are expressed or not

We analyzed two cohorts of liver cancer patients, a previously described cohort from Mongolia of 70 HCC patients [27], and a new cohort from Thailand comprised of iCCA (100/147, 68.0 %) and HCC (47/147, 32.0 %). Cohort characteristics are summarized in **Methods**, Table 1, and Fig. 2. Whole genome RNASeq data were collected from pair tumor and non-tumor samples as described previously (**Methods**) [27].

### AAV segments are preferentially detectable in non-tumor RNASeq samples

**Fig. 3:**
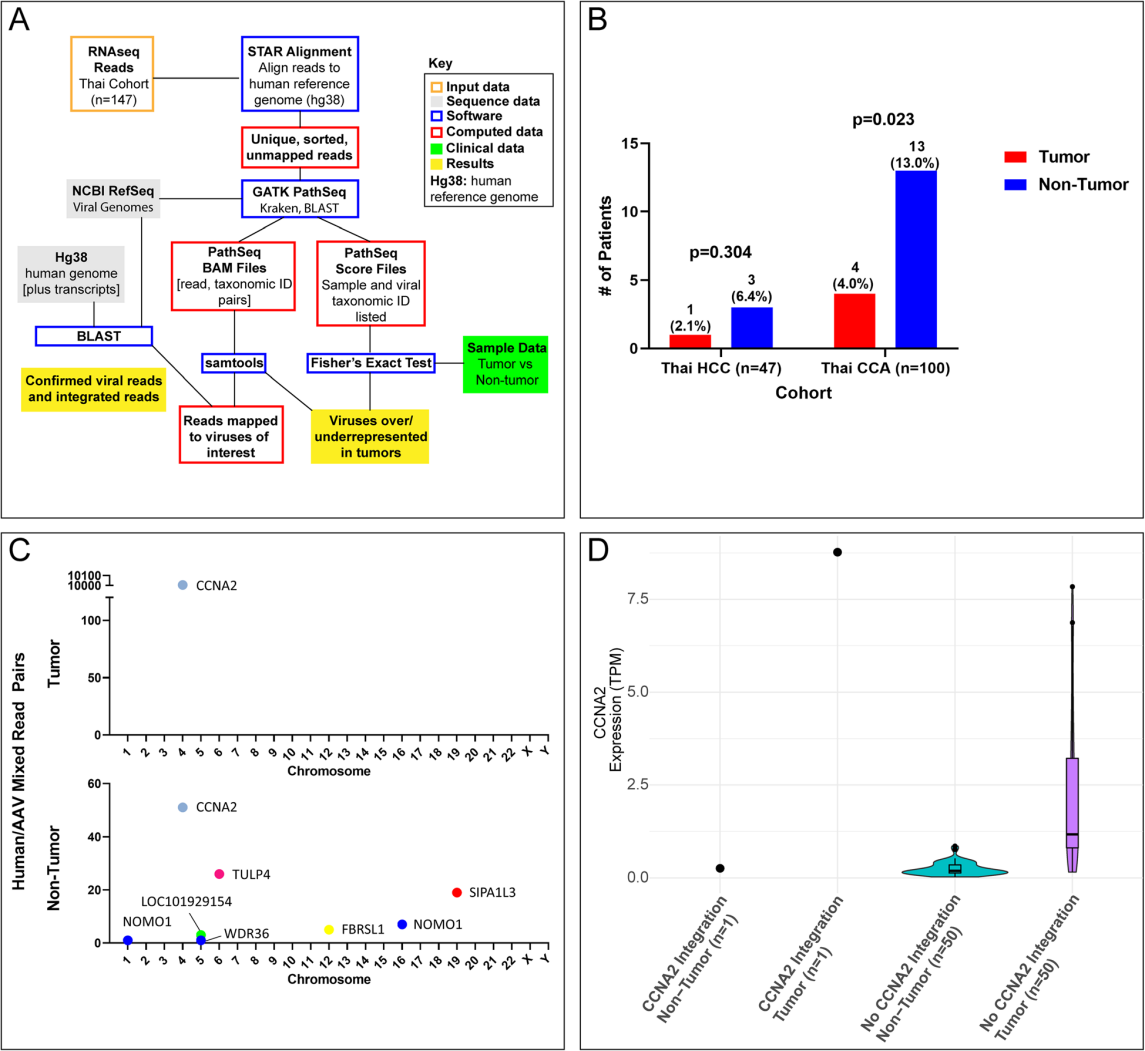
**A** Workflow of RNAseq analysis (**B**) AAV2/13 detected in Thai patients with HCC and iCCA (**C**) Counts of host genome integrations of AAV2/13 in tumor vs. non-tumor tissue in viral capture DNAseq samples (**D**) RNA expression of *CCNA2* measured in transcripts per million (TPM) by AAV2/13 integration status in tumor vs. non-tumor

As described in **Methods** and Fig. 3 A, we sought sequence evidence of non-hepatitis virus infections occurring preferentially in tumor or in non-tumor samples. The only non-hepatitis virus genus that was significantly differentially present between non-tumor samples and tumor samples was *Dependoparvovirus*, which was detected by PathSeq [28] in 17/147 non-tumor samples and 0/147 tumor samples (*p* < 9.4 × 10^−6^, Fisher’s exact test). The best matching strain was not necessarily AAV2; two samples with reads matching AAV2 also had at least one read whose best PathSeq match was AAV3, with the caveat that the PathSeq library did not include the partial genome AAV13. All reads identified by PathSeq as matching to AAV were confirmed by blastn [29]. We found two samples with at least one chimeric read indicating an integration into a gene (Supplementary Table S1). We also found reads from AAV present in two of the Mongolian non-tumor samples.

### Further analysis of AAV segments and host genome integration with viral capture DNASeq

To confirm and expand the evidence of AAV genomic segments being present, we did viral capture DNASeq (**Methods**) on the tumor and non-tumor samples from the 19 patients who showed evidence of AAV presence in the RNASeq analysis. Evidence of AAV integration into the host genome is more likely to be detected via viral capture DNASeq because it enriches for viral sequences and because AAV may integrate anywhere in the host genome, not only in expressed genes.

By sequence analysis (**Methods**) of the DNA data, we confirmed AAV presence in all 17 Thai non-tumor samples, but not in the two Mongolian non-tumor samples (Supplementary Table S1). AAV presence was also detected in 5/17 Thai tumor samples. Thus, in the Thai data, presence of AAV in the RNA of a non-tumor sample was a perfect predictor of presence of AAV in the DNAseq data. However, absence of AAV in the RNAseq data is not a perfect predictor of absence in the DNAseq data because (i) the DNA sequence may not be transcribed and (ii) because of the limitations of PathSeq discussed below in the case of 719T. Partitioning by tumor type, the enrichment of AAV in tumor tissue compared to non-tumor tissue in HCC (*n* = 1,2.1 % vs. *n* = 3,6.4 %, *p* = 0.304) was not statistically significant, but in iCCA, the predominance of AAV-present samples among the non-tumor samples (*n* = 13,13.0 %) vs. tumor tissue (*n* = 4, 4.0 %), was significant (*p* = 0.023) (Fig. 3B).

**Fig. 4:**
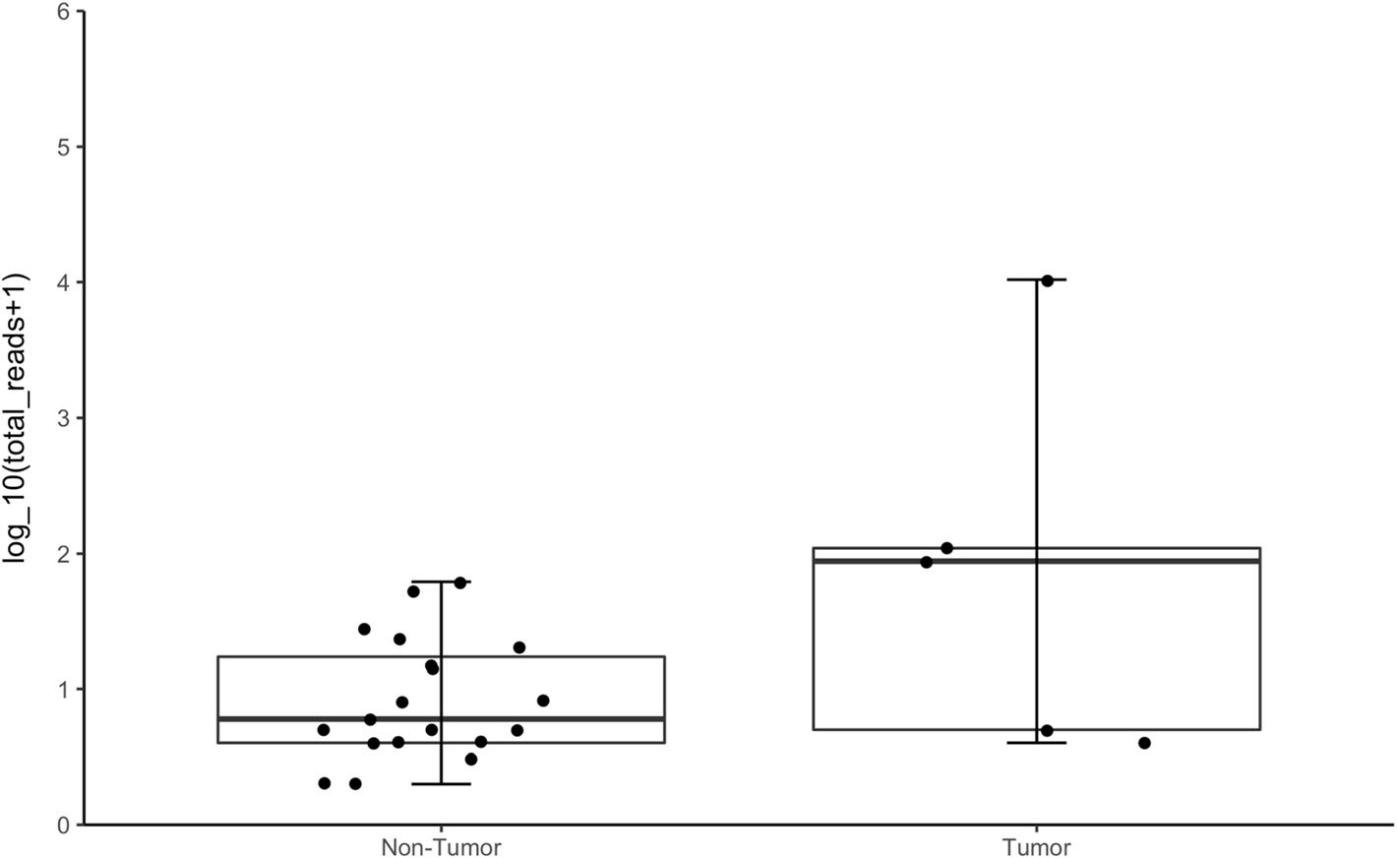
The distribution of chimeric reads in the samples that have at least one AAV integration. These boxplots visualize part of the tabular data in Supplementary Table S1

Next, we explored AAV2/13 integration among the 17 Thai patients with AAV present. We did not detect any tumor-specific enrichment of AAV2/13 integrations; most integrations were in the non-tumor samples (Supplementary Table S1). There was only one AAV2/13 integration into an established target gene for AAV-mediated oncogenesis, namely *CCNA2*, in an HCC patient (719T). This integration was found with identical breakpoints in both tumor and non-tumor tissue (719T and 719NT). All other AAV integrations were exclusively in one of the two paired samples of each patient (Supplementary Table S1). In patient 719, there was increased mRNA expression of *CCNA2* compared to patients lacking the integration (Fig. 3 C-D*)*. The proportion of patients with AAV (13 %) is significantly lower (Fisher’s exact test, two-sided *P* < 0.005) compared to the 21 % reported in the largest European study 5. Aside from the geographic contribution to this disparity, difference in AAV positivity may also be due to differences in ascertainment, such as the tumor types and the proportion of cases with cirrhosis. Only two Thai iCCA patients have AAV integrations in the tumor sample, each with two distinct integrations (776T and 779T; Supplementary Table S1). Figure 4 visualizes the numbers of chimeric reads in each sample that are shown in tabular form in Table S1. None of the four integrations were detected in the corresponding non-tumor sample. Three of the four integrations are not in or near a human gene and one occurs in the gene *PRKCB*, which has not been previously implicated and does not show increased expression. Hence, we conclude that the iCCA AAV integrations are not oncogenic in these two Asian cohorts.

One limitation of our integration site analysis is that for the purposes of cost efficiency and focus, our study design applied DNA sequencing only in samples from those patients where we could find presence of AAV in the RNAseq data. We could have missed many DNA integrations of AAV into intergenic regions of the host genomes of other patient samples. Indeed, we found several intergenic integrations of AAV (Supplementary Table S1) in the DNA samples we analyzed, but these seem to be functionally irrelevant. The proposed mechanism(s) of oncogenesis induced by AAV integration always include(s) altered transcription of a human gene (near) where the AAV integration occurs. Therefore, we believe that requiring AAV to be present in the RNA is a reasonable precondition to search for functionally relevant AAV integrations in the DNA host genome.

Most of the 147 patients were tested for hepatitis B virus (HBV) infection status by the standard surface antigen (HBsAG) (Table 1). By this criterion (see CDC_HepatitisB_SerologicTest_FactSheet9.indd), the HBV-tested patients with AAV integrations are all HBV negative.

### Reanalysis of data from one patient for whom the initial RNASeq analysis and the DNASeq analysis seemed to give conflicting results

There was an apparent discrepancy between the analysis of the RNASeq data and the DNASeq data of patient 719 who has the oncogenic integration of AAV in the gene *CCNA2*. In the RNASeq data and using PathSeq [28], we found only AAV segments present, but no evidence of integration, and only in the non-tumor sample. To address the discrepancy, we did more targeted analysis of the RNASeq data (**Methods**). The AAV-targeted analysis revealed five human/AAV chimeric reads in the tumor sample having the same breakpoint in *CCNA2* as detected in the DNASeq data. These were missed by PathSeq because the AAV pieces are too small to form a seed for the alignment part of PathSeq.

### Resolving published discrepancies and extending analyses of AAV integration in TCGA samples

We revisited the question of AAV integrations in TCGA liver cancer (LIHC and CHOL) samples because of the discrepant results of previous studies summarized in **Background**. We applied our PathSeq-centered [28] pipeline (Fig. 3 A) to 462 RNASeq samples from 404 TCGA patients; for 397/404 patients self-reported race and/or ethnicity information is available in TCGA. We validated any possible matches with more targeted sequence analysis (**Methods**). The 462 TCGA samples comprise 371 primary HCC (LIHC) samples, 50 matched normal samples from HCC patients, 33 primary CCA (CHOL, which are mostly intrahepatic CCA [30] like our Thai CCA samples) samples, and 8 matched normal samples from iCCA patients.

PathSeq found genus *Dependoparvovirus* in 6/58 non-tumor samples and 6/404 tumor samples, which is a significant difference in proportions (*P* < 0.00077, Fisher’s exact test one-sided). All the matches found by PathSeq in genus *Dependoparvovirus* were to AAV, but not always to AAV2. The tumor sample BD-A2L6 had a match to AAV7 in the PathSeq analysis without a match to AAV2. All 12 of these matches were validated by searching for AAV-matching reads with SRPRISM and ReadAligner [31] and by aligning the read pairs found by SRPRISM to all seven AAV sequences and the human genome with blastn [29] using the most sensitive word size of 11.

The results of the prior studies on TCGA and ours are compared in Supplementary Table S2. Except for one discrepancy with the integration site on TCGA sample TCGA-BC-A10T[4], we found all the previously reported instances of AAV presence [4, 5] and AAV integration. We also found six previously unreported samples with AAV segments present without integration and two non-tumor samples with integrations that support our positional analysis of AAV integrations described below. None of the 12 samples with an AAV integration come from an Asian patient (*P* < 0.0023, Fisher’s exact test, one sided, based on 161 Asian, 236 non-Asian, 7 not classified) supporting our conclusion that oncogenic integrations of AAV are very rare in Asian liver cancer patients. The TCGA samples classified as Asian are not classified more precisely by country of origin.

There are at least four technical challenges in searching for AAV in TCGA. First, the TCGA liver cancer read lengths are only 48 nucleotides. Second, the matches may be to AAV strains different from AAV2 or even different from any AAV strain with a complete genome [3]. Third, the TCGA samples were not subjected to viral capture sequencing, so even one read mapping at high identity to AAV is unexpected and interesting. Fourth, as shown previously [3, 4, 11, 16], when AAV does integrate, only a piece of the viral genome is detectable and it may be present at high copy number, as we observed in our sample 719T. The overarching reason for the false negatives in two previous studies [11, 12] is that they were taxonomically broad, intending to find all viruses using homogeneous methods and assumptions that are not necessarily correct for AAV. In contrast, we, and Bayard et al. [4] focused our search on AAV and used methods that are more suitable for this taxon.

### Positional analysis of oncogenic and non-oncogenic AAV integrations

Based on analysis of the AAV locations of integrated segments in our samples, TCGA, and previously published samples, we propose that the oncogenic potential of the AAV integrated pieces depends on (i) the AAV genome interval retained and (ii) the integration site in the human genome [3–5]. The initial findings that AAV integration can be oncogenic [3] were disputed [32–34] because (i) other studies had suggested that AAV infection may protect against cancer [34, 35], (ii) the observed rate of oncogenic AAV integrations was very small compared to the prevalence of AAV infection [33] and (iii) more patients had non-oncogenic AAV integrations [3, 32]. We similarly observed non-oncogenic integrations in the non-tumor samples of 10 Thai patients. Another heretofore unexplained finding is that all published oncogenic integrations are of strains resembling AAV2/13[3, 5], but one of the non-oncogenic integrations in TCGA is of AAV7.

**Fig. 5:**
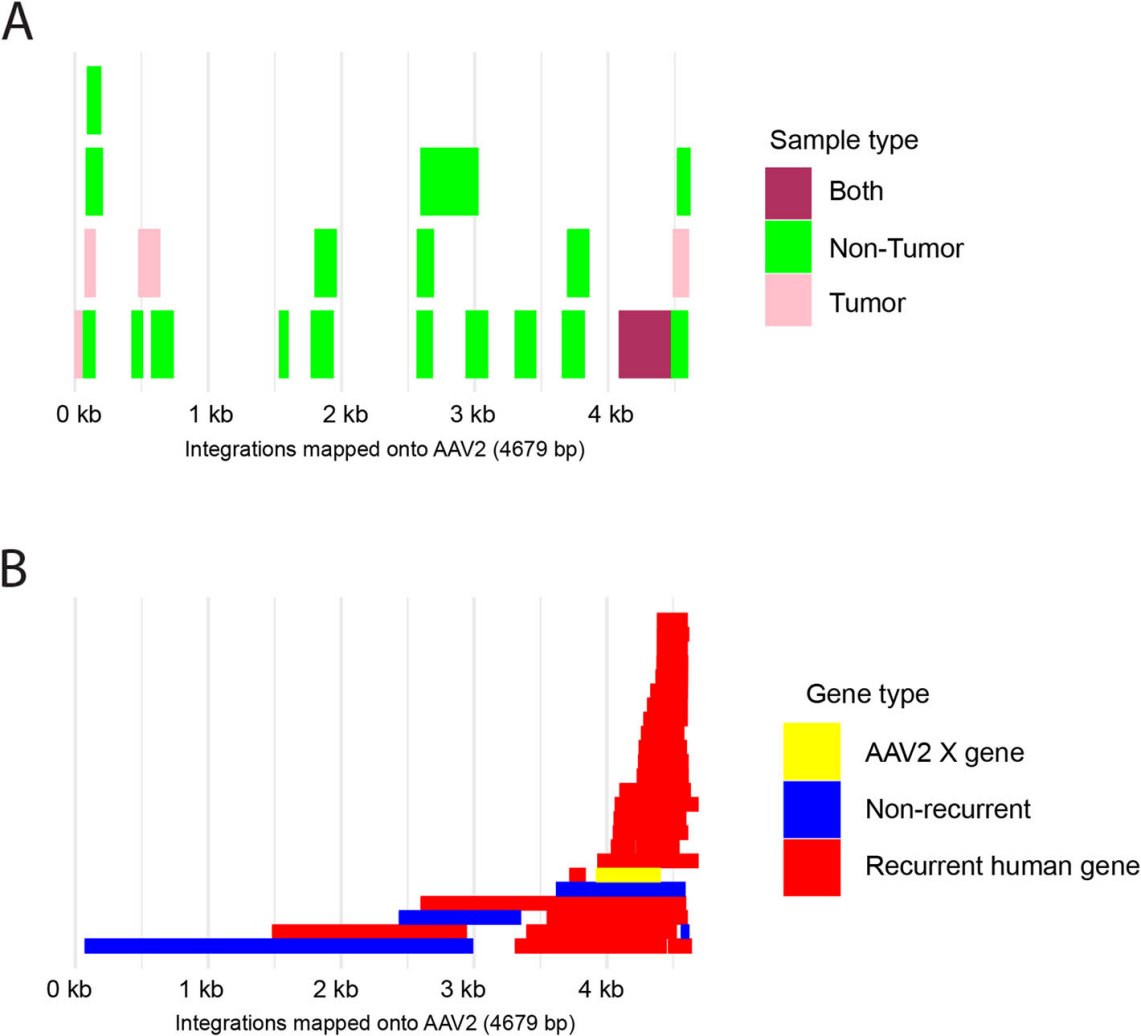
Comparison of AAV integrations into new Thai samples and published AAV integrations with respect to the AAV2 genome as the reference. In both panels, the X axis represents the base pair positions in the AAV2 reference genome and overlapping integrated segments are stacked on top of one another; there is no Y axis, but the number of rectangles stacked on top of one another indicate the number of overlapping integrations from that region of AAV. **A** Thirteen out of 17 Thai patients with samples subjected to viral capture sequencing had at least one integration of AAV. Ten had integrations only in the NT sample; two had integrations only in the T sample. Only one patient had a possibly clonal integration into the gene *CCNA2*. That integration, shown in maroon, is the only one that overlaps the X gene. **B** AAV intervals spanned by previously published integrations of AAV into liver cancer samples. The data for panel **B** are adapted from the supplementary information of reference 5; see also our Supplementary Table S3

We posit that the X gene of AAV2, encoded at positions 3,929-4,396 near the 3’ end of the virus genome, strengthens its oncogenic potential [14, 15]. The early experiments on the X gene suggest that its presence may enhance AAV’s capability to replicate and that its presence can endow AAV genomes with the capability to transform infected cells towards a cancerous state. Twenty-four out of 26 of the previously published likely oncogenic integrations [5] (bad line breakSupplementary Table S3, upper part) that had both boundaries overlapping the X gene, while 2/4 likely non-oncogenic AAV integrations (Supplementary Table S3, lower part) do not overlap the X gene (Fig. 5). All the oncogenic integrations in TCGA overlap the X gene (Supplementary Table S3). Among the seven fully sequenced strains of AAV, only the AAV2 strain contains an intact X gene, which could explain the strain bias among the observed oncogenic integrations.

The (full-length) X-protein is the following 155 amino acid protein (YP_009110690.1).

MVLYLPTSREATDKQLPQMSTHKAFFQAWSGRTEMCTFRGPSGQRFHTRTDIFTPLPSWVDSDLNTLLHRFSSRTPRYLRILRPPSVRQSLLPSSHSTPRDRSAWRSSGSCRRKTANAGIPKFSTLPTTTSLLMWTLLWTLMACIQSLAPLAPDT.

Cao et al. suggested that there may be shorter isoforms that are suffixes of the above sequence starting from one of the other two methionines among the first 50 amino acids [22]. A search of the protein non-redundant (nr) database with X protein as query found only one significant match (E-value 0.0002 to sequence QDX47270.1, which is the 760aa capsid protein of another AAV found in the Chinese bat species *Rhinolophus pusillus* [36]. Interestingly a BLASTP search [29] with QDX47270.1 as query finds many capsid proteins from AAV sequences aligning within the interval [1.435] of QDX47270. However, if one restricts the search to the C-terminal portion [436.760] of QDX47270.1, then only the match to the AAV2 X protein has a significant E-value (0.0004); the slight differences in the E-values are because interchanging query and subject in BLAST preserves alignments, but not E-values.

Our hypothesis positionally complements the previous findings that the interval 4,402-4,534, adjacent to the X gene, contains several sequences to which liver-specific transcription factors such as HNF1-α and HNF6 can bind [37]. This notion is positionally consistent with a previous hypothesis that AAV integrations are almost always from the 3’ half of the genome to avoid overlap with the replication (Rep) gene products, ending at position 2,252, because the Rep proteins are anti-oncogenic [3, 37]. These findings [37] explain the liver-tropism and why 24/26 previously published potentially oncogenic integrations into recurrent genes (Supplementary Table S3, Fig. 5) intersect the AAV interval 4,402-4,534. However, they do not explain the AAV2 strain-specificity and why most integration intervals extend several hundred nucleotides in the 5’ direction from position 4,402 into the X gene interval. Our hypothesis, that if the AAV integrated piece overlaps the X gene then its oncogenic potential is increased, would explain the strain specificity and the positional data.

## Conclusions

In summary, our analysis of 217 Thai and Mongolian patients found that AAV2/13 integration into known oncogenes is rare. In contrast to European patients, it is not only rare, but there is minimal evidence of its contribution to oncogenesis in HCC and iCCA. We also found that among TCGA liver cancer samples, all samples with AAV present are non-Asian. These findings broaden previous findings that rates of AAV integration in Japanese and Korean HCC patients are low [6, 38]. A recent study of 413 cancer patients in China, including 49 HCC patients, found a high rate (~ 80 %) of presence of AAV, but no evidence of oncogenic integration [18]. The contrast in evidence for the oncogenic potential between European and Asian patients suggests epidemiological differences allowing AAV2/13 to drive oncogenesis in European patients. Future studies should focus on epidemiological and environmental exposure differences between Asian, American, European and African countries to elicit potential interactions of geography and presence of AAV in HCC etiology.

Lastly, by sequence analysis of previously reported and newly identified AAV integrations, we corrected the AAV integrations in TCGA (Supplementary Table S2) and we suggested a new hypothesis that the role of AAV2 X gene could explain why some AAV integrations predispose to liver cancer and some do not.

## Methods

### Patients

The study included patients from two cohorts from Thailand and Mongolia. The Thai cohort contained 147 liver cancer patients, 47 with HCC and 100 with iCCA. These are a subset of 171 patients from the TIGER-LC Consortium of which an earlier cohort was previously described [39]. All patients gave informed consent. The study was approved by the Institutional Review Boards of the respective institutions (NCI protocol number 13CN089; CRI protocol number 18/2555; Chulabhorn Hospital protocol number 11/2553; Thai NCI protocol number EC163/2010; Chiang Mai University protocol number TIGER-LC; Khon Kaen University protocol number HE541099). The original 171 patients were reduced to 147 because of: (i) lack of suitable samples for RNA sequencing (ii) poor tumor quality (iii) missing clinical data (iv) inconclusive pathology data on the tumor type.

The second cohort consisted of 70 patients with HCC from Mongolia. These patients are a subset of the 76 patients described previously with available RNA sequencing data available for analysis [9]. The study was approved by the ethics committee at the National Cancer Center in Ulaanbaatar, Mongolia.

We used the Consort tool to prepare a flow chart, Fig. 2, summarizing the patient selection and some key analysis steps.

### RNA sequencing

RNA sequencing of the Mongolian was described previously [27] and RNA sequencing of the Thai cohort was done similarly. For each patient, RNASeq data were generated from a tumor sample and a nearby, matched non-tumor sample. Almost all reads had lengths in the range 51–150 after trimming adapters.

### Analysis of RNA sequencing data

To search for viruses in the RNASeq data we used (GATK) PathSeq [28] in the version of February 2019. We specified as the host genome the hg38 version of the human genome that was current as of February 6, 2019. For possible pathogens, we used a library of all 10,532 viral RefSeq genomes that was also current on the same date; all other parameters to PathSeq used default settings. This library includes the seven full AAV genomes mentioned above but not AAV13.

To run PathSeq, a key precursor step is to align the RNA reads to the hist genome, so that reads mapping to the host genome can be filtered out as not being candidates to align to a pathogen genome. For that purpose, we used STAR [40] with the following settings:

--peOverlapNbasesMin 10 --alignEndsProtrude 10 ConcordantPair --outSAMunmapped Within --outFilterType Normal --outFilterMultimapNmax 10 --outFilterMismatchNmax 10
--outFilterMismatchNoverLmax 0.3 --alignIntronMin 21 --alignIntronMax 0
--alignMatesGapMax 0 --limitSjdbInsertNsj 1,000,000 --sjdbGTFfile my.gtf
--alignSJoverhangMin 5 --alignSJDBoverhangMin 3 --sjdbScore 2
--outFilterMatchNminOverLread 0.66 --quantMode TranscriptomeSAM --outReadsUnmapped Fastx --outSAMattributes All

The AAV matches found by PathSeq were validated with Kraken [41] also using the same library of virus genomes and with blastn [21] using a reference database comprised of just AAV genomes. As explained below, we reanalyzed the data from one patient with more targeted tools because PathSeq can miss microbial matches when: (i) the read is chimeric human/microbe) and the microbial part is too close to one end or (ii) the sequence has N characters or (iii) the sequence matches a species or strain that is not in the database of RefSeq genomes.

### Viral capture DNA sequencing

For the 19 (17 Thai, 2 Mongolian) patients who had evidence of AAV presence in the RNA sequencing data analysis, we submitted both the cancer and non-cancer samples (total of 38 samples) for viral capture DNA sequencing with an Agilent (Santa Clara, CA, USA) custom capture kit. The kit included a tiled array of overlapping 120mer probes spaced 15nt apart and starting at positions 1, 16, 31, 46, 61, 76,… of the AAV sequences listed above. For AAV2 our sequence probes were of the same length and density as in the seminal paper [3]. Some probes from regions of the human genome where Nault et al. found AAV integrations were used as controls. They did not use any other strains in their capture design, but they did compare against other AAV strains in their sequence analysis because some mismatches are tolerated between probe and bait in viral capture sequencing. We used other strains because we wanted to understand to what extent the AAV segments seen in liver cancers are exclusively from strains resembling AAV2. A recent study of 413 cancer samples of nine types including HCC, tested for 13 different strains (a.k.a., serotypes) and found presence of AAV1, AAV2, a hybrid of AAV2 and 3, AAV8 and AAVrh.43[18].

The sequencing was done on an Illumina (San Diego, CA) NovaSeq sequencer using S1 chips in the NCI Frederick sequencing facility. The sequence reads in our DNA sequencing assay are paired end reads with each mate having length 151nt. The total number of reads per sample ranged between 85 million and 106 million. In all 38 samples, more than 90 % of bases had a quality score of at least 30. Samples were pooled for sequencing, which has the consequence that a small number of reads get assigned to the wrong sample via a phenomenon called “barcode hopping” or “index hopping”. We corrected for this problem as described below.

### Evidence for AAV presence and integration in viral capture DNASeq

**Fig. 6:**
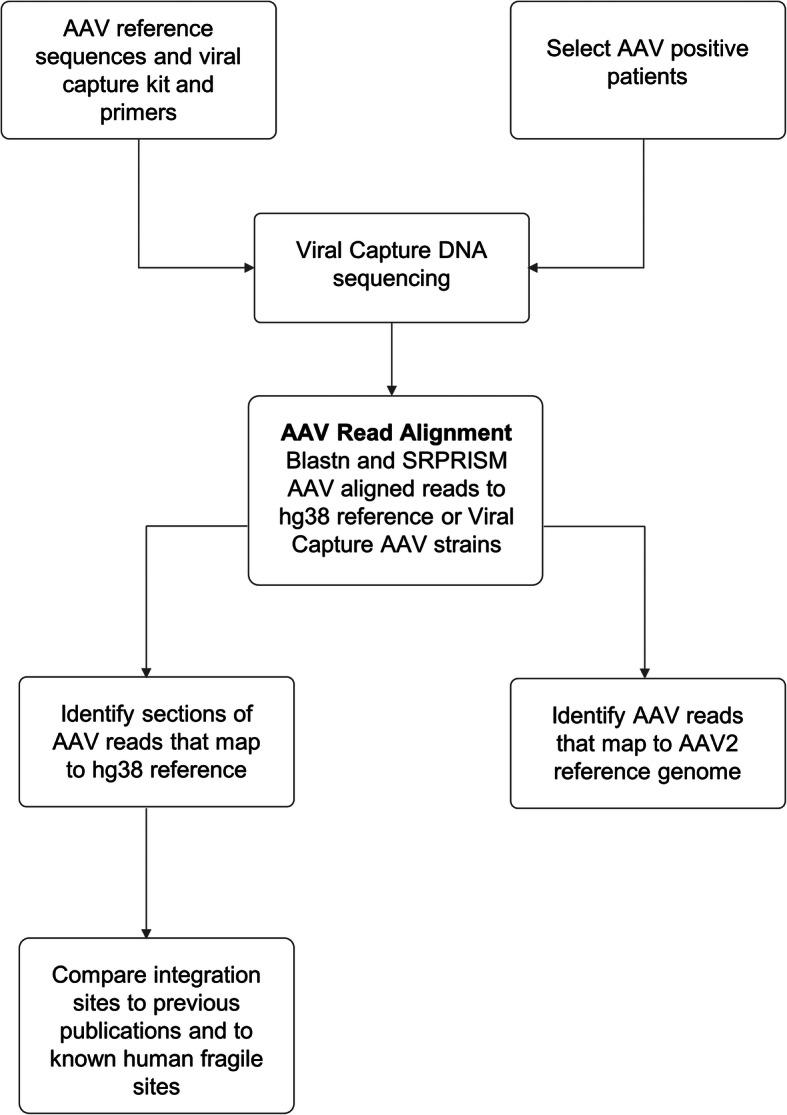
Flowchart describing viral capture DNA sequencing and its analysis

The steps of DNA sequencing and analysis are described in a flowchart in Fig. 6.

Viral capture sequencing generates paired reads with spacing of hundreds of (not sequenced) nucleotides in between. Consequently, there are two kinds of evidence of viral integration. The weaker evidence consists of mate pairs R1 and R2, such that one read is entirely from the human genome and the other read is entirely from the virus genome; we call these “cross-species read pairs”. The stronger evidence consists of one read that has a substantial piece from the human genome and a substantial piece from the virus genome. We call these “chimeric reads”. A “substantial piece” is one long enough to generate a statistically significant match in a blastn search [29]; in practice the minimum size is between 11nt and 28nt depending on what database size and other blastn parameters one uses. Chimeric reads are stronger evidence than “cross-species pairs” because chimeric reads make it possible to pinpoint the integration boundary, with the potential for only a few nucleotides of ambiguity. When the only available evidence consists of “paired reads” the virus boundaries are unknown [5]. Sometimes all the chimeric reads have the virus on the same end and hence one can determine one out of two integration boundaries [5].

### Sequence analysis of viral capture DNA data and for reanalysis of RNASeq data

We used SRPRISM and a streamlined version called Readfinder [31] and blastn [29] to identify reads that mapped either to any of the AAV strains used to design the Viral Capture sequencing kit and/or to the human genome. As explained in the previous subsection, we are interested in mate pairs in which at least one read maps to AAV and at least one read maps to the human genome and the two mapping tests require separate runs of SRPRISM/Readfinder using either the AAV genomes or the human genome as the reference genome(s). SRPRISM, unlike other more commonly used read aligners, provides guarantees on the number of mismatches tolerated. These guarantees are important because (i) the available whole genomes for AAV are few and (ii) the sequence data of previously reported AAV integrations (GenBank sequences submitted in conjunction with reference 4) do not match perfectly to the reference genome for AAV2 (NC_001401.2). In our usage, the difference between SRPRISM and Readfinder is in parameter setting, but the matches found by both packages are identical. Readfinder can find reads that have high-quality alignments to a genome, such as AAV, over part of the read with default settings, while for SRPRISM one must specify in what subinterval of the read one expects to find the match. For blastn we used word sizes (i.e., the size of the initial seed) of 11 or 16, which are lower than the default of 28 because we expected that some chimeric reads would by chance have the breakpoint between the AAV segment and the human segment occurring within 16nt of either end of the 151nt read.

For example, a pair of Readfinder commands to compare all reads against all AAV genomes would look like.

readfinder --log-file test1.log --db AAV_genomes -i sample1_mate1.fastq --in-fmt fastq –output sample1_mate1.fa -B 31

readfinder --log-file test1.log --db AAV_genomes -i sample1_mate2.fastq --in-fmt fastq –output sample1_mate2.fa -B 31

The outputs are FASTA files (suffix fa) with reads that have a sufficiently long match. We varied the -B parameter, which determines how many nucleotides need to be matched, between 20 and 31. Separate Readfinder commands were run for each sample and each set of read mates.

A blastn command to compare a DNAseq read against the AAV genomes would look like:

blastn -query oneread.fa -db AAV -word_size 11 -out oneread_vs_AAV.blastout

where the name of the query file and output file vary.

For reads that mapped only to the human genome or only to one of the AAV strains, we retained only reads that had alignments of at least 140nt and 96 % identity. For reads that mapped to both organisms, we imposed no filters on alignment quality since these are the likely chimeric reads.

We developed in-house python programs (i) to identify cross-species mate pairs in which one mate has a high-quality alignment to the human genome and the other mate has a high-quality alignment to AAV and (ii) to identify chimeric reads and (iii) to define how much of the AAV2 genome was covered by reads mapping to AAV. We defined a read to be “chimeric” if it has alignments to both the human genome and AAV that together span at least 140nt of the 151nt read and overlap by at most 6nt. We also developed an in-house python program to summarize the coverage in the AAV genome by a set of reads in a sample, which we measured as the number or proportion of nucleotides of NC_001401.2 covered by at least two reads in the sample. The searches for cross-species mate pairs and chimeric reads were done after removing a few reads that were likely assigned to the wrong sample, as described in the next subsection.

We defined that a sample had AAV present based on the viral capture sequence if at least 10 read mates map to AAV and they cover at least 10 % of the AAV2 genome. Integrations of AAV into patients DNASeq samples are shown in **Table S1.** This table shows only those samples with AAV integrations, but not the other five patients who had evidence of AAV presence in the RNASeq data.

### Removal of reads likely assigned to the incorrect sample via “barcode hopping”

One known limitation of multiplexed Illumina sequencing is that reads are sometimes assigned to the wrong sample via a phenomenon known as “barcode hopping” or “index hopping”. Although this problem affects only a small (< 1 %) proportion of reads, this can be a serious problem when one is trying to make absolute assertions about the presence or absence of a rare object in a sample. In our study, the misassignment of AAV reads to a sample *S*, could lead us to conclude incorrectly that AAV is present in *S*. We reasoned that a read *r* is likely to be misassigned to sample *S1*, if *r* occurs exactly one time in the reads for *S1 *and multiple times in a different sample *S2* (*). For “multiple times”, we used the threshold 2, but almost all examples of reads meeting the two-part criterion (*) in our data satisfied the higher threshold of at least 5 reads assigned to sample *S2*. Using a combination of unix commands and in-house programs in perl and python applied to all reads mapping to AAV, we identified and removed 71 read pairs in which at least one read met the criterion (*).

## Supplementary information

Additional file 1 **Table S1.** AAV presence and integrations in paired Thai non-tumor (NT) and tumor (T) samples. (Excel)

Additional file 2 **Table S2. **Reanalysis of AAV presence and integration events in TCGA samples (Excel).

Additional file 3 **Table S3.** Positional analysis of published AAV integrations.

## Data Availability

The reads mapping to AAV and associated metadata are available via National Center for Biotechnology Information (NCBI) repositories starting from the Bioprojects database and identifiers PRJNA729528 (Thailand) and PRJNA603277 (Mongolia). These Bioprojects database identifiers are linked to other identifiers in the NCBI Biosamples database for information on the study subjects and in the NCBI Sequence Read Archive (SRA) for the reads mapping to AAV.
